# Differences in kinetic characteristics during countermovement jump of football players with cerebral palsy according to impairment profiles

**DOI:** 10.3389/fphys.2023.1121652

**Published:** 2023-04-26

**Authors:** Matías Henríquez, Rafael Sabido, David Barbado, Alba Roldan, Jose L. L. Elvira, Javier Yanci, Raúl Reina

**Affiliations:** ^1^ Sports Research Centre, Department of Sport Sciences, Miguel Hernández University, Elche, Spain; ^2^ Escuela de Kinesiología, Facultad de Odontología y Ciencias de la Rehabilitación, Universidad San Sebastián, Providencia, Chile; ^3^ Alicante Institute for Health and Biomedical Research (ISABIAL), Alicante, Spain; ^4^ Society, Sports, and Physical Exercise Research Group (GIKAFIT), Physical Education and Sport Department, Faculty of Education and Sport, University of the Basque Country (UPV/EHU), Vitoria-Gasteiz, Spain

**Keywords:** force, jumping, brain injury, concentric, Paralympic

## Abstract

**Objectives:** The purpose of this study was 1) to determine and compare kinetic parameters during the realization of a countermovement jump (CMJ) between footballers with cerebral palsy (CP) and non-impaired footballers, and 2) to analyze the differences in this action between different players’ impairment profiles and a group of non-impaired footballers.

**Methods:** This study involved 154 participants comprising 121 male footballers with CP from 11 national teams and 33 male non-impaired football players recruited as the control group (CG). The footballers with CP were described according to the different impairment profiles (bilateral spasticity = 10; athetosis or ataxia = 16; unilateral spasticity = 77; minimum impairment = 18). All participants performed three CMJs on a force platform to record kinetic parameters during the test.

**Results:** The group of para-footballers presented significantly lower values than the CG in the jump height (*p* < 0.01, *d* = −1.28), peak power (*p* < 0.01, *d* = −0.84), and the net concentric impulse (*p* < 0.01, *d* = −0.86). Concerning the pairwise comparisons between CP profiles and the CG, significant differences were found for the bilateral spasticity, athetosis or ataxia, and unilateral spasticity subgroups compared to the non-impaired players for jump height (*p* < 0.01; *d* = −1.31 to −2.61), power output (*p* < 0.05; *d* = −0.77 to −1.66), and concentric impulse of the CMJ (*p* < 0.01; *d* = −0.86 to −1.97). When comparing the minimum impairment subgroup with the CG, only significant differences were found for jump height (*p* = 0.036; *d* = −0.82). Footballers with minimum impairment presented higher jumping height (*p* = 0.002; *d* = −1.32) and concentric impulse (*p* = 0.029; *d* = −1.08) compared to those with bilateral spasticity. Also, the unilateral spasticity subgroup reports a higher jump height performance than the bilateral group (*p* = 0.012; *d* = −1.12).

**Conclusion:** These results suggest that the variables related to power production during the concentric phase of the jump are crucial for the performance differences between groups with and without impairment. This study provides a more comprehensive understanding of kinetic variables that would differentiate CP and non-impaired footballers. However, more studies are necessary to clarify which parameters better differentiate among different profiles of CP. The findings could help to prescribe effective physical training programs and support the classifier’s decision-making for class allocation in this para-sport.

## 1 Introduction

Vertical jump assessment, in the form of the countermovement jump (CMJ) test, is broadly used for the measurement of neuromuscular status (Claudino et al., 2017), lower-limb muscle power (Cormie et al., 2009), fatigue assessment (Brownstein et al., 2017), training load optimization (Claudino et al., 2012), and is also considered a relevant index for talent identification in football players (Stølen et al., 2005; Castagna and Castellini, 2013). Football requires repetitive actions based on stretch-shortening cycles in different physical and technical situations such as jumping (Bangsbo, 2014). Hence, the jumping ability of football players is an aspect that has been extensively studied (Stølen et al., 2005). This ability is frequently assessed through the CMJ test, as it is an easy test to evaluate and can provide a high number of relevant variables for performance monitoring [i.e., power/force development, rate of force development (RFD), velocity, eccentric time/concentric time, jump height]. In fact, some researchers have demonstrated the relationship between CMJ parameters and sprint performance, kicking velocity, and maximal strength in the lower extremities, supporting the relevance of certain variables in mechanical power output (Wisløff et al., 2004; Dobbs et al., 2015; Rodríguez-Lorenzo et al., 2016).

The vertical jump capacity is also included in the evaluation of football players with cerebral palsy (CP) as a form of monitoring physical training objectives (Yanci et al., 2016) and for classification purposes to define who can compete in this para-sport (Yanci et al., 2016; Reina et al., 2018). CP football is a discipline similar to the regular sport of intermittent characteristics, presenting some adaptations (i.e., played 7-a-side, 50 m × 70 m field size, 5 m × 2 m goals, no-offside rule, among others) and bringing the possibility to be practiced by people with neurological conditions (Yanci et al., 2016). Because of the variety of motor impairments presented by football players with CP, only those with an eligible impairment of hypertonia, ataxia, or athetosis are eligible to practice this para-sport (International Paralympic Committee, 2016). Players with CP present verifiable physical problems that affect key activities for sports performance, presenting different severity of the impairments according to the body involvement such as hemiplegia, with one side of the body affected (e.g., more affected upper limb than the lower limb); diplegia affecting all limbs but with higher impairments in lower limbs than in upper limbs; or quadriplegia, with involvement in four limbs and trunk (Graham et al., 2016). In CP football, players with a minimum impairment level can participate in CP football competitions, being relevant to know the differences with able-bodied footballers and identifying if the eligible impairment impacts the sports performance of high functional participants, which is constantly a challenge for the classification process (Tweedy et al., 2018). Some of the principal neurological characteristics and secondary consequences of players’ muscle function are related to the presence of overstretched sarcomere lengths, increments in the passive mechanical stiffness, changes in the extracellular matrix, and transformation of fiber type due to the consequences of the damage in cortical pathways in the central nervous system (Mathewson and Lieber, 2015; Graham et al., 2016; Dayanidhi and Lieber, 2018) possibly having a direct effect on the performance of motor actions related to football demands, like jump performance (Reina et al., 2018; Reina et al., 2019). In this regard, Yanci et al. (2016) also reported a lower anaerobic performance reflected in vertical jump capability compared to competitive and amateur footballers without disabilities.

The CMJ test has shown good reliability (ICC = 0.88) and validity for assessing jump performance in footballers with CP (Reina et al., 2018). However, studies that have investigated this issue are scarce in CP football and some of the results are inconclusive. For example, Yanci et al. (2016), found no relationship between CMJ height performance and the classification profile of a team with 12 Spanish footballers with CP. Conversely, Reina et al. (2018) showed significant differences in the same performance variable between functional profiles in a group of 132 international para-footballers, revealing differences according to impairment profile. With regard to this approach, the limited analysis of the jump height and peak power performance may overlook the complex nature of CMJ and the different neuromuscular strategies used by players of eligible motor involvement for CP football (Gathercole et al., 2015). Therefore, a more comprehensive understanding of the key variables that differentiate between impairment profiles and contribute to performance in the vertical jump may enable the prescription of effective physical training programs for improving strength and conditioning practice in the athletic population with CP. On the other hand, the identification of key jump parameters could potentially favor the development of practical implications in evidence-based classification, improving the strategies to support the class allocation of para-footballers with neurological impairments. To the best of the authors’ knowledge, there is limited information regarding kinetic variables in the vertical jump performance of footballers with CP, with studies generally only focused on jump height performance (Reina et al., 2018), peak power output (Yanci et al., 2016), and vertical ground reaction force (Cámara et al., 2013). However, no previous research has investigated kinetic variables during CMJ according to the different impairment profiles presented in football players with CP. Therefore, this study aimed 1) to determine and compare kinetic parameters during the realization of a CMJ between international footballers with CP and non-impaired footballers, and 2) to analyze the differences in this action between the different players’ impairment profiles [e.g., bilateral spasticity (BS), athetosis/ataxia (AA), unilateral spasticity (US), and minimum impairment (MI)] and non-impaired footballers.

## 2 Methods

### 2.1 Participants

A convenience sample of 154 participants comprising 121 male international footballers with CP from 11 national teams and 33 male non-impaired football players [i.e., control group (CG)] volunteered to participate in this study. The para-footballers were described according to the different impairment profiles, such as BS (i.e., spastic diplegia), AA (i.e., coordination impairments), US (i.e., spastic hemiplegia), and MI (i.e., mild impairment regarding the other three profiles) based on the description of eligible impairments or affected limbs included in the International Federation of Cerebral Palsy Football classification rulebook (IFCPF, 2018) and the Cerebral Palsy International Sports and Recreation Association (CPISRA) classification system (Reina, 2014) that stills applicable in Paralympic sports such as Para Athletics (World Para Athletics, 2022). Players of the MI group are those who presented an eligible impairment of hypertonia (i.e., spasticity grade 1-2 in at least one muscle group), ataxia (i.e., clear signs of cerebellar dysfunction with tremor or dysmetria in coordination tests), or dyskinesia (i.e., athetoid or dystonic movements in different parts of the body, affecting balance and coordination) that meets the minimum impairment severity requirement to be eligible to play CP football (Reina et al., 2021a). All the participants presented a functional profile to be categorized in Level 1 of the Gross Motor Function Classification System (Jahnsen et al., 2006). The inclusion criteria for participating in the study were to be eligible to compete in this para-sport (i.e., presenting the minimum eligible impairment of hypertonia, ataxia, or athetosis), have a valid license from the IFCPF, be at an international level competing at world championships, and not present any injuries in the 3 months before data collection. In the case of the CG, a group with equivalent years of football training experience was selected. The general characteristics of the participants are described in Table 1. Before the testing session, players received detailed verbal instructions and were fully familiarized with the jumping procedure in which the CMJ test was part of the sport classification process. The study’s characteristics were described to the participants, detailed in an oral explanation and they provided written informed consent in concordance with Helsinki’s declaration (2013). The human ethics committee of Miguel Hernández University approved all the study procedures (Reference number DPS.RRV.01.14).

**TABLE 1:** Participants’ characteristics data.

Participant groups	n	Age (years)	Body mass (kg)	Height (cm)	BMI (kg·m^−2^)	Training experience (years)
Cerebral palsy group	121	25.7 ± 6.6	72.1 ± 10.3	175.6 ± 7.4	23.4 ± 3.0	10.4 ± 7.5
Bilateral spasticity	10	25.7 ± 5.9	74.2 ± 8.2	177.3 ± 6.9	23.6 ± 2.5	11.1 ± 5.2
Athetosis/Ataxia	16	26.3 ± 7.4	72.8 ± 9.8	174.9 ± 6.4	23.6 ± 2.8	9.6 ± 4.1
Unilateral spasticity	77	25.2 ± 6.0	70.6 ± 10.4	175.2 ± 7.5	23.1 ± 3.2	9.3 ± 6.7
Minimum impairment	18	27.7 ± 8.6	76.9 ± 10.2	177.3 ± 8.3	24.5 ± 2.9	15.3 ± 11.4
Control group	33	19.6 ± 4.0	76.7 ± 8.5	179.1 ± 6.2	24.2 ± 2.3	9.8 ± 5.6
Overall sample	154	24.7 ± 6.6	73.1 ± 10.0	176.1 ± 7.3	23.5 ± 2.9	10.3 ± 7.1

Data are expressed as mean and standard deviation. BMI, body mass index.

### 2.2 Procedures

A cross-sectional study was conducted to examine kinetic variables during the CMJ, considering the impairment profiles of the footballers with CP and comparing them with the CG. Before the testing, all the players were required to avoid strenuous physical activity. A standardized warm-up was performed, consisting of a 5-min self-paced low-intensity run, skipping exercises, strides, and two 15-m sprints with and without changes of direction (Reina et al., 2018). The players were asked to perform three CMJs with 30 s of recovery between each trial in a single session. The highest height reached in any of the attempts was considered for subsequent analyses of the selected kinetic variables.

### 2.3 Measures

#### 2.3.1 Vertical jump capacity

To perform the CMJ, the participant started in an upright standing position on the force platform with fixed hands on hips and sustained that position during the jump execution. All participants performed a fast flexion downward movement to reach approximately 90° of knee flexion, followed by performing a maximum vertical jump with hip and knee extension and plantar flexion during the flight phase and landing in the starting place, trying to reach the maximal height in every trial. Those para-athletes who presented spastic hemiplegia and difficulty maintaining their hand on their hip were allowed to keep their hand on one side of their body, keeping the extremity static during the jump (Yanci et al., 2016). Performance variables were evaluated on a force platform (Kistler™, Switzerland, Model 9287B) that recorded vertical forces with a sampling rate of 1,000 Hz. Custom software designed in LabView (version 12.0, National Instruments, Texas, United States) was used to analyze the CMJ test outcome variables. For jump testing, the jump height (JH; units: cm) maximum displacement was calculated with the following formula V_t_^2^/2*g*, where V_t_ is the velocity at take-off (m/s) instant and *g* is gravity acceleration (9.8 m/s^2^). The velocity was obtained from the integral force with respect to time using the trapezoidal rule (Linthorne, 2001), and the highest jump being considered for subsequent analyses. The peak power output (PPO; units: W/kg) was calculated as the product of the vertical velocity and force data during the CMJ. The eccentric phase of the jump was considered when velocity was negative and included the quiet phase, unweighting phase, and braking phase from the start of the movement to zero velocity (Cormie et al., 2009). The onset of the movement was identified 30 ms before the instant when vertical force was equal to or more than five times the standard deviation of body weight which is calculated in the weighing phase (McMahon et al., 2018). Variables assessed in the eccentric phase were the peak force eccentric (PFE; units: N/kg), the mechanical impulse quantified as the total force applied during the eccentric phase and normalized by body mass (EIMP; units: N·s·kg^−1^), and the time to peak of the eccentric force (TTPEF; units: s). The concentric phase includes the propulsive stage before the takeoff, where the velocity was positive in the force-time curve. The peak force concentric (PFC; units: N/kg) was the maximal force achieved during the concentric phase where the change in displacement was positive and vertical, and the net force concentric impulse (CIMP; units: N·s·kg^−1^). Additionally, time to peak concentric force (TTCF; units: s) was considered. Each jumping phase’s rate of force production was quantified using the maximum rate of force development (mRFD; units: N/s), which was defined as the largest force increase during a 20 ms epoch (Woodard et al., 1999). In addition, the time to the maximum rate of force development (TTmRFD; units: s) was also computed (Reina et al., 2019). Time of the total jump duration (JD; units: s), deceleration phase (DP; units: s), eccentric phase duration (ED; units: s), concentric phase duration (CD; units: s), and the rate of eccentric and concentric impulse (E/C; units: rate) were also registered during the CMJ. All the variables of force and power were relativized by the participant’s body mass (Cormie et al., 2009).

### 2.4 Statistical analysis

The results are presented as means and standard deviation (±). The data distribution and homogeneity of variances were assessed with Kolmogorov–Smirnov and Levene’s tests, respectively. The coefficient of variation [(CV), in %] was calculated within groups using the following formula: CV= (*SD/Mean*·100) (Atkinson and Nevill, 1998). An independent or unpaired Student’s *t*-test was used to determine the magnitude of differences between the group of footballers with CP and the CG on the kinetic CMJ test variables. Considering the characteristics of the data and the different groups, a one-way analysis of variance (ANOVA) with Tukey’s *post hoc* analysis was conducted to determine which kinetic variables of CMJ performance differs according to the different impairment subgroups of footballers with CP, including the CG. Cohen’s *d* index effect size (ES) was considered following the interpretation proposed by Rhea (2004) for highly trained athletes: above 1.00, between 0.50 and 1.00, between 0.25 and 0.50, and lower than 0.25 were considered large, moderate, small, and trivial, respectively. In addition, the Hedges *g* index (Hedges and Olkin, 1985) was used to provide an ES estimation reducing the bias caused by small samples (n < 20). The analysis was performed with the Statistical Package for Social Science (version 26, SPSS Inc., Chicago, IL, United States) and GraphPad Prism (Prism v6.0, San Diego, CA, United States). Statistical significance was set as *p* < 0.05.

## 3 Results

### 3.1 Comparison between cerebral palsy group and control group

Table 2 presents the differences in CMJ parameters between para-footballers with CP and the CG, including the ES and the CV. The unpaired *t*-test showed that JH performance in the group of para-footballers reported significantly lower values than the CG (*p* < 0.01; *d* = −1.28, large). Additionally, PPO (*p* < 0.001; *d* = −0.84, large), and CIMP (*p* < 0.001; *d* = −0.86, large), were also significantly lower in the CP football group compared to the CG.

**TABLE 2:** Performance variables of a countermovement jump for cerebral palsy football players and control group.

CMJ variables	Overall sample		CP group		Control group		*p*	*d*
	M ± SD	CV (%)	M ± SD	CV (%)	M ± SD	CV (%)		
Jump height (cm)	27.4 ± 6.4	23.4	25.8 ± 5.9	22.9	33.1 ± 4.9	14.8	<0.001**	−1.28
Jump duration (s)	0.99 ± 0.33	33.3	1.00 ± 0.36	36.0	0.96 ± 0.16	16.7	0.513	0.12
Time to the maximum RFD (s)	0.68 ± 0.35	51.5	0.69 ± 0.38	55.1	0.64 ± 0.21	32.8	0.400	0.14
Maximum RFD (N/s)	8,475 ± 4,018	47.4	8,445 ± 4,161	49.3	8,587 ± 3,501	40.8	0.858	−0.04
Peak power output (W/kg)	23.35 ± 5.10	21.8	22.48 ± 4.90	21.8	26.54 ± 4.61	17.4	<0.001**	−0.84
Eccentric/Concentric impulse rate	0.24 ± 0.08	33.3	0.24 ± 0.08	33.3	0.24 ± 0.06	25.0	0.872	0
Eccentric phase								
Deceleration phase (s)	0.68 ± 0.22	32.4	0.68 ± 0.23	33.8	0.68 ± 0.14	20.6	0.864	0
Eccentric duration (s)	0.43 ± 0.13	30.2	0.42 ± 0.13	31.0	0.45 ± 0.13	28.9	0.338	−0.23
Time to peak eccentric force (s)	0.26 ± 0.09	34.6	0.26 ± 0.10	38.5	0.25 ± 0.09	36.0	0.922	0.1
Peak force eccentric (N/kg)	3.60 ± 1.90	52.8	3.58 ± 2.01	56.1	3.68 ± 1.44	39.1	0.806	0.05
Eccentric impulse (N·s·kg^−1^)	0.76 ± 0.31	40.8	0.74 ± 0.32	43.4	0.83 ± 0.26	30.8	0.130	−0.29
Concentric Phase								
Concentric duration (s)	0.56 ± 0.32	57.1	0.58 ± 0.35	60.3	0.51 ± 0.13	25.5	0.283	0.22
Time to peak concentric force (s)	0.82 ± 0.34	41.5	0.82 ± 0.37	45.1	0.79 ± 0.16	20.3	0.678	0.09
Peak force concentric (N/kg)	13.09 ± 2.75	21.0	13.03 ± 2.93	22.5	13.28 ± 1.96	14.8	0.655	−0.09
Concentric Impulse (N·s·kg^−1^)	3.06 ± 0.49	15.9	2.98 ± 0.49	16.3	3.38 ± 0.34	10.1	<0.001**	−0.86

The Student’s *t*-test for independent measures. CMJ, countermovement jump; CP, cerebral palsy, M, mean; SD, standard deviation; CV, coefficient of variation; ES, effect size; RFD, rate of force development. Significant differences between players groups in CMJ variables (**p < 0.01).

### 3.2 Comparison between cerebral palsy impairment profiles

Table 3 displays the vertical jump performance variables of a CMJ according to para-footballers’ impairment profiles and the CG. Overall, the one-way ANOVA analysis showed significant differences between the functional profiles in the data variables of JH, PPO, and CIMP (*p* < 0.01). However, there were no significant differences in the rest of the variables (*p* > 0.05). The multiple between-subgroups comparisons for the force-time characteristics’ values reveal that the CG showed significantly higher scores, with moderate-to-large ESs, compared to BS, AA, and US impairment profiles for the variables JH (*p* < 0.01; *d* = −1.31 to −2.61), PPO (*p* < 0.05; *d* = −0.77 to −1.66), and CIMP (*p* < 0.01; *d* = −0.86 to −1.97).

**TABLE 3:** One-way analysis of variance for performance variables differences of a countermovement jump according to cerebral palsy impairment profiles and control group.

CMJ variables	Bilateral spasticity *n* = 10		Athetosis/Ataxia *n* = 16		Unilateral spasticity *n* = 77		Minimum impairment *n* = 18		Control group *n* = 33
	M ± SD	CV (%)	M ± SD	CV (%)	M ± SD	CV (%)	M ± SD	CV (%)	M ± SD
Jump height (cm)	20.2 ± 4.7	23.4**	24.6 ± 6.1	24.6**	26.2 ± 5.4	20.5**^†^	28.5 ± 6.7	23.6*^††^	33.1 ± 4.9
Jump duration (s)	0.98 ± 0.13	13.6	0.93 ± 0.29	31.4	1.04 ± 0.42	40.2	0.93 ± 0.17	17.8	0.96 ± 0.16
Time to the maximum RFD (s)	0.71 ± 0.15	20.9	0.63 ± 0.27	42.5	0.71 ± 0.45	62.5	0.66 ± 0.23	35.0	0.64 ± 0.21
Maximum RFD (N/s)	8,085 ± 3,605	44.6	7,954 ± 2,690	33.8	8,546 ± 4,504	52.7	8,648 ± 4,226	48.9	8,587 ± 3,501
Peak power output (W/kg)	18.75 ± 4.63	24.7**	22.33 ± 4.63	20.7*	22.78 ± 4.95	21.7**	23.43 ± 4.46	19.0	26.54 ± 4.61
Eccentric/Concentric impulse rate	0.25 ± 0.05	20.6	0.23 ± 0.06	25.6	0.24 ± 0.09	39.3	0.26 ± 0.06	23.1	0.24 ± 0.06
Eccentric phase									
Deceleration phase (s)	0.70 ± 0.09	13.0	0.67 ± 0.23	34.3	0.69 ± 0.26	37.9	0.66 ± 0.13	20.4	0.68 ± 0.14
Eccentric duration (s)	0.45 ± 0.06	14.2	0.46 ± 0.16	35.83	0.41 ± 0.14	34.8	0.42 ± 0.09	20.3	0.45 ± 0.13
Time to peak eccentric force (s)	0.28 ± 0.07	25.9	0.28 ± 0.14	48.7	0.25 ± 0.10	40.3	0.25 ± 0.10	38.3	0.25 ± 0.09
Peak force eccentric (N/kg)	2.90 ± 1.02	35.2	3.27 ± 1.77	54.3	3.63 ± 2.22	61.1	4.05 ± 1.63	40.4	3.68 ± 1.44
Eccentric impulse (N·s·kg^−1^)	0.69 ± 0.22	31.6	0.66 ± 0.23	34.8	0.74 ± 0.35	47.5	0.84 ± 0.29	34.4	0.83 ± 0.26
Concentric phase									
Concentric duration (s)	0.53 ± 0.12	22.8	0.47 ± 0.18	38.8	0.62 ± 0.42	67.7	0.51 ± 0.12	23.6	0.51 ± 0.13
Time to peak concentric force (s)	0.80 ± 0.13	16.3	0.78 ± 0.30	38.9	0.85 ± 0.43	50.9	0.73 ± 0.19	26.2	0.79 ± 0.16
Peak force concentric (N/kg)	12.33 ± 2.69	21.8	13.40 ± 3.08	23.0	13.04 ± 3.01	23.1	13.06 ± 2.77	21.2	13.28 ± 1.96
Concentric impulse (N·s·kg^−1^)	2.67 ± 0.40	14.9**	2.83 ± 0.42	14.9**	3.00 ± 0.48	16.1**	3.19 ± 0.50	15.7^†^	3.38 ± 0.34

CMJ, countermovement jump; M, mean; SD, standard deviation; RFD, rate of force development. *p < 0.05, **p < 0.01, Significant differences with respect to the control group. ^†^ *p* < 0.05, ^††^ *p* < 0.01, Significant differences with respect to the bilateral spasticity group.

Table 4 shows the results obtained in the comparison between the groups of players with CP and the CG. In JH, pairwise comparisons revealed that the BS group obtained lower values than the US and MI profiles during the CMJ test (*p* < 0.012; *d* = −1.12 to −1.32, large). Additionally, significant and moderate ES was found for the comparison between the MI group and the CG (*p* = 0.036; *d* = −0.82). Although the differences found were not significant, moderate to large ESs were found for JH between BS and AA groups comparisons (*p* = 0.272; *d* = 0.78), and for PPO between BS and AA (*p* = 0.344; *d* = −0.75), the BS and US (*p* = 0.093; *d* = −0.81), and BS and MI (*p* = 0.099; *d* = −1.01) subgroups.

**TABLE 4:** Pairwise comparison in the performance variables of a countermovement jump according to cerebral palsy impairment profiles and control group.

CMJ variables	BS vs. AA		BS vs. US		BS vs. MI		BS vs. CG		AA vs. US		AA vs. MI		AA vs. CG		US vs. MI		US vs. CG		MI vs. CG	
	*p*	*d*	*p*	*d*	*p*	*d*	*p*	*d*	*p*	*d*	*p*	*d*	*p*	*d*	*p*	*d*	*p*	*d*	*p*	*d*
Jump height	0.272	0.78	0.012	−1.12	0.002	−1.32	0.001	−2.61	0.829	−0.29	0.232	−0.59	0.001	−1.60	0.481	−0.41	0.001	−1.31	0.036	−0.82
Jump duration	0.993	0.20	0.990	−0.15	0.995	0.31	1.000	0.13	0.745	−0.27	1.000	0	0.997	−0.14	0.750	0.28	0.797	0.22	0.999	−0.18
Time to the maximum RFD	0.986	0.33	1.000	0	0.998	0.24	0.983	0.35	0.923	−0.19	0.999	−0.12	1.000	−0.04	0.986	0.12	0.837	0.18	0.999	0.09
Maximum RFD	1.000	0.04	0.997	−0.1	0.997	−0.14	0.997	−0.14	0.984	−0.14	0.988	−0.19	0.986	−0.19	1.000	−0.02	1.000	−0.01	1.000	0.02
Peak power output	0.344	−0.75	0.093	−0.81	0.099	−1.01	0.001	−1.66	0.997	−0.09	0.961	−0.24	0.034	−0.91	0.985	−0.13	0.002	−0.77	0.176	−0.67
Eccentric/Concentric impulse rate	0.921	0.34	0.971	0.11	1.000	−0.17	0.995	0.17	0.991	−0.12	0.793	−0.5	0.967	−0.16	0.856	−0.23	0.997	0	0.967	0.33
Eccentric phase																				
Deceleration phase	0.992	0.15	1.000	0.04	0.985	0.33	0.997	0.15	0.991	−0.08	1.000	0.05	0.997	−0.06	0.975	0.12	0.977	0.04	0.998	−0.14
Eccentric duration	1.000	−0.07	0.907	0.3	0.971	0.36	1.000	0	0.786	0.35	0.938	0.31	1.000	0.07	1.000	−0.07	0.720	−0.29	0.949	−0.25
Time to peak eccentric force	1.000	0	0.872	0.31	0.920	0.32	0.937	0.34	0.857	0.28	0.927	0.24	0.942	0.27	1.000	0	1.000	0	1.000	0
Peak force eccentric	0.989	−0.23	0.784	−0.34	0.548	−0.77	0.791	−0.56	0.958	−0.17	0.758	−0.45	0.955	−0.26	0.920	−0.2	1.000	−0.02	0.964	−0.24
Eccentric impulse	0.999	0.13	0.984	−0.15	0.720	−0.54	0.687	−0.55	0.830	−0.24	0.401	−0.67	0.316	−0.67	0.750	−0.29	0.624	−0.28	1.000	0.04
Concentric phase																				
Concentric duration	0.990	0.36	0.911	−0.22	1.000	0.16	1.000	0.15	0.417	−0.38	0.995	−0.26	0.994	−0.27	0.680	0.28	0.446	0.31	1.000	0
Time to peak concentric force	1.000	0.08	0.992	−0.12	0.985	0.4	1.000	0.06	0.923	−0.17	0.996	0.2	1.000	−0.05	0.654	0.3	0.914	0.16	0.973	−0.35
Peak force concentric	0.875	−0.35	0.939	−0.24	0.962	−0.26	0.878	−0.44	0.991	0.12	0.997	0.11	1.000	0.05	1.000	−0.01	0.994	−0.09	0.999	−0.10
Concentric impulse	0.886	−0.38	0.188	−0.7	0.029	−1.08	0.001	−1.97	0.676	−0.36	0.147	−0.76	0.001	−1.47	0.473	−0.39	0.001	−0.86	0.605	−0.46

CMJ, countermovement jump; BS, bilateral spasticity; AA, athetosis/ataxia; US, unilateral spasticity; MI, minimum impairment; CG, control group, *d*, index effect size; RFD, rate of force development.

For the PFE, the MI and CG subgroups exhibit no significant differences with moderate ES compared to players from the BS subgroup (*p* > 0.791; *d* = −0.56 to −0.77, moderate). No significant differences were obtained in the pairwise comparison for EIMP and PFC (*p* > 0.05). However, a moderate ES was found between BS and MI (*d* = −0.54), BS and CG (*d* = −0.55), AA and MI (*d* = −0.67), and AA and CG (*d* = −0.67) in EIMP. With regard to CIMP, pairwise comparisons reported higher scores for the para-footballers of the MI subgroup compared to those of the BS subgroup (*p* = 0.029; *d* = −1.08, large), and moderate ES without significant differences compared to the AA subgroup (*p* = 0.147; *d* = −0.76, moderate).

No significant differences were reported for the between-group comparisons in mRFD (*p* > 0.05; *d* = 0.04 to 0.19, trivial). During braking forces, AA and US had higher coefficients of variation in the variables PFE and EIMP. Additionally, para-footballers with AA and hemiplegic profiles reported the highest CV in the duration of the eccentric phase during CMJ.

The descriptive data regarding the force-time curve of the CMJ according to para-footballers’ impairment profiles and non-impaired footballers throughout the movement are described in Supplementary Figure S1.

## 4 Discussion

The present study aimed to determine and compare kinetic parameters during the realization of a CMJ in football players with and without brain impairments and to analyze the differences among players with different profiles/severity of impairment and non-impaired footballers. This study showed that power output in concentric variables associated with force and velocity capabilities of the lower limbs discriminate between footballers with CP and non-impaired footballers (i.e., JH, PPO, and CIMP variables). Regarding the differences among impairment profiles and CG, the heights reached during the vertical jump, peak power values, and concentric impulse were significantly different compared to BS, AA, and US; but the MI subgroup only performed lower JH compared to non-impaired players. Moreover, those players with higher functionality presented higher values compared to those with BS in JH and CIMP variables. Additionally, athletes with a hemiplegic profile (US) presented higher jump height performance than the BS subgroup.

### 4.1 Comparisons between cerebral palsy group and control group

Differences in the JH performance between footballers with CP and the CG could be explained by the fact that this variable is primarily affected by the net vertical impulse during the concentric phase, permitting the body mass displacement at a higher velocity (Kirby et al., 2011; McMahon et al., 2017). Previous research on non-impaired participants has revealed that the mechanical power output and net vertical propulsive variable were differentiating factors for CMJ height between sexes (McMahon et al., 2017), sport-specific practice (Laffaye et al., 2014), and age (Louder et al., 2018; Alvero-Cruz et al., 2021). The results of this study are consistent with the study of Yanci et al. (2016) on a small group of para-footballers, who presented lower peak power compared to amateur and elite players without brain damage. Similarly, Reina et al. (2018) found that mainstream football players realized higher vertical jumps in comparison to players with CP with different functional profiles, categorized according to their impairment severity and body involvement. Moreover, Fleeton et al. (2022) reported a significant relationship between the achieved height and the concentric relative impulse in athletes with CP, supporting the relevance to the overall performance in the CMJ. Thus, performance differences between para-footballers’ subgroups could be attributed to the characteristics presented in the population with CP, such as diminished muscle size (O’Brien et al., 2021), impaired neuromuscular activation (Rose and McGill, 2005), or higher coactivation patterns (Hussain et al., 2014), impacting on the performance of functional activities.

The eccentric contraction during the CMJ is an essential component that contributes to propulsive forces in the stretch-shortening cycle (McBride et al., 2008; Turner and Jeffreys, 2010; Gathercole et al., 2015). During the eccentric phase of the jump, the unweighting component impacts the force production during the braking and propulsive phases, which is also conditioned by movement patterns and para-footballers’ strategies to decelerate the body (McMahon et al., 2018). This study found no significant differences between groups in variables related to the eccentric phase, probably because the CP showed great variability of functional patterns due to the nature of the diagnosis itself (Graham et al., 2016). Our results reinforce the idea that the concentric force generation was more critical in achieving height during the vertical jump. Para-footballers with CP could use different strategies during the jump descending phase according to each individual’s neurological impairment, maybe reflecting the consequences of muscle weakness, altered coordination, muscular co-contraction, and/or muscular spasticity during downward movements in the CMJ (Reina et al., 2018).

It should be noted that in previous studies, the RFD was identified as an index of relevant explosive strength due to the significant functional implications in motor function and the influence during the vertical jump proficiency in conjunction with the neuromuscular coordination of the lower extremities (Rodacki et al., 2002; McLellan et al., 2011; Maffiuletti et al., 2016). However, previous studies showed that children and adults with CP presented a reduced RFD compared to non-impaired individuals, principally using lower limb isometric testing to explore the associations with impaired mobility (Moreau et al., 2012; Geertsen et al., 2015; Goudriaan et al., 2018). The present RFD values showed no significant differences, suggesting that this parameter did not discriminate between the CP group and the CG. Results indicate that non-impaired footballers reached a higher JH, principally producing a greater concentric impulse, irrespective of the RFD variable. The interpretation of these results requires caution due to the multiple factors that affect RFD production (i.e., physiological and methodological factors) (Rodríguez-Rosell et al., 2018) and the high degree of response variability presented in this study.

### 4.2 Comparison between cerebral palsy impairment profiles

Concerning the kinetic variables, differences were only found for JH, PPO, and CIMP when comparing BS, AA, and US subgroups with CG, and for JH when comparing MI with CG. In addition, jump height performance showed differences between players’ profiles, which is similar to what has been reported in a previous study on footballers with CP and different impairment profiles (Reina et al., 2018). Coinciding with these results, Antunes et al. (2017) reported that para-athletes with CP and profiles of BS and AA presented the smallest performance values during vertical jumps. Those players with a more impaired profile, such as BS, showed the lowest score in the JH performance than the more functional groups (i.e., US and MI), probably due to differences in muscle power production and the vertical concentric impulse. The players with BS or spastic diplegia present clinical characteristics associated with motor disorders that are more pronounced in both lower limbs (Graham et al., 2016), limitations in the range of movement of ankle dorsiflexion with variable loss of hamstring length (Kilgour et al., 2005), spasticity presence as the most common dominant motor disorder (Rethlefsen et al., 2010), and impaired muscle strength attributable to deficits in voluntary activation associated with constraints in gross motor parameters (Ross and Engsberg, 2007; Eek et al., 2011). These factors could probably affect the lower power output during the jump, which depends on velocity and limits the voluntary contractile capacity of explosive muscle force production (González-Badillo and Marques, 2010; Earp et al., 2011).

In this study, the participants with AA (i.e., ataxia or athetosis) impairments presented a tendency of difference with moderate ES in terms of the variables of JH (i.e., vs. BS and MI), PPO (i.e., vs. BS and CG), eccentric (i.e., vs. MI and CG), and concentric impulse (i.e., vs. MI). Players with AA are characterized by presenting difficulties with impaired voluntary control due to ataxia or involuntary contractions due to the athetosis, compromising the orderly muscular sequencing, and problems in realization of movements with aberrant force, rhythm, and inaccuracy, which impact football-specific skills (Reina et al., 2021b). Possibly, these coordination impairments influence the downward movement required during the eccentric jump phase, where mechanical changes in technique contribute to CMJ performance. The CMJ test requires the capacity to effectively transfer the stretch-shortening cycle incrementing the eccentric force-velocity parameters translating momentum to concentric force, which involve complex interactions of multi-joint movements, musculotendinous units, and neuromuscular factors that contribute to the performance (Cormie et al., 2011; Thomas et al., 2015). Involuntary sustained or intermittent contractions, tremors, and dysmetria, among other hyperkinetic movement disorders commonly presented in footballers with CP profiles, may influence the transfer momentum between jump phases and the contribution of the eccentric force impulse production (Sanger et al., 2010). Nevertheless, future studies should focus on identifying jumping strategies and the constraints produced by hyperkinetic movements.

When comparing the player groups with the participants who presented a hemiplegic profile, significant differences and considerable ES were only found with the BS group and CG in the variables JH, PPO, and the magnitude of concentric vertical impulse during the jump. Accordingly, para-footballers with hemiplegic profiles present marked asymmetries when performing a vertical jump, as a consequence of muscle weakness and motor compensation strategies influenced by the impairment characteristics (Runciman et al., 2016; Reina et al., 2019). Hussain et al. (2014) analyzed the neuromuscular factors contributing to muscle weakness in isometric contractions of 11 active sportsmen with spastic hemiplegia, showing that the paretic limb presents an impaired neural activation in conjunction with differences in the gastrocnemius anatomical cross-sectional area. The essential contribution of neuromuscular factors and the stretch-shortening cycle capabilities as determinants of vertical jump performance is well known, and it might be expected that these characteristics also impact the vertical jump performance parameters. However, in the case of para-athletes with CP, even having impaired components of these determinants, a higher training background could provide greater adaptations enhancing physical proficiency (Runciman et al., 2016; McMahon et al., 2018).

Players with MI showed magnitude differences and moderate to large effect sizes compared to BS and AA in variables related to JH, power output production, and force/velocity parameters during both phases of the vertical jump. These results are consistent with early studies showing that players with mild impairment profiles present a higher physical capacity and perform better than those with more impaired profiles in different activity limitation assessments (Reina et al., 2018; Reina et al., 2020). The kinematic jump results could be linked due to the characteristics of this group, which include mild manifestations of players with bilateral spasticity, coordination impairments, and unilateral spasticity, characterized by the presence of the minimum impairment criteria (Peña-González et al., 2021). The MI group presented only significant differences in the height achieved during the vertical jump, however, in the rest of the variables, only a moderate ES was found in the power-related parameter. This group characteristically presents a minimal impact of the neurological consequences produced by CP on activity limitation, so it might appear that the values obtained were close to those obtained by the non-impaired players, suggesting possible adaptations due to high-level training (Runciman et al., 2016).

The results of the present study should be considered with caution due to the unequal number of participants in each subgroup, and the limited number of differences found between impairment profiles in the group of footballers with CP. However, it should be noted the difficulties to recruit participants with these neurological characteristics. Hence, the study of the jumping characteristics in participants with specific involvement such as bilateral spasticity, ataxia, and dyskinesia warrants further investigation to deeper understand the relationship between impairment and relevant physical features.

## 5 Conclusion

Based on the authors’ knowledge, this is the first study to determine differences in jump parameters between footballers with CP according to their impairment profiles and taking into consideration the eccentric/concentric phases of a vertical jump, suggesting that the variables related to power production during the concentric phase of the jump are crucial for the performance differences between individuals’ with and without impairment. Considering the variable JH, only significant differences were found between BS and US, BS and MI, BS and CG, AA and CG, US and CG, and MI and CG. Moreover, regarding the kinetic variables and the pairwise comparisons, only significant differences were found for the power output between BS and CG, AA and CG, US and CG; and for the concentric impulse in BS and MI, BS and CG, AA and CG, US and CG. However, a limitation of this study was that playing positions and subgroup analysis through CMJ performance variables (e.g., low- and high-skill jumpers) or individual differences were not considered. Additionally, the limited number of participants in some profiles (i.e., BS and AA) and the over-representation of participants with unilateral spasticity, which is a characteristic of CP football (Reina et al., 2021a), may reduce the statistical power analysis. Further studies should consider incorporating a control for the downward movement and a temporal-phase analysis to identify differences along with the entire CMJ force and power time parameters and elucidate which factors are more determinant to provide a deeper understanding of the different profiles presented in footballers with CP. In addition, the relationships between the characteristics of the spastic muscle structure components and relevant stretch-shortening cycle movements such as vertical jumps should be considered.

In terms of the practical implications of this study, strength and conditioning coaches should include in their programs training routines focus on power muscle actions that improve those parameters of vertical jump performance and replicate the most frequent challenging actions of matches to try to compensate for the negative impairment consequences on the motor actions. On the other hand, classifiers must consider in the decision-making for the class allocation that lower-limb concentric parameters are the key factor that differentiates between groups with and without impairment during CMJ performance, and which depends on the neuromuscular capacity of players with CP. In other words, classification procedures would not be focused only on the jumping (i.e., jump height); including some variables related to kinetic and kinematics may better reflect the relationships between eligible impairment and activity limitation.

## Data Availability

The raw data supporting the conclusion of this article will be made available by the authors, without undue reservation.
