# Supplementary material for: Differences in kinetic characteristics during countermovement jump of football players with cerebral palsy according to impairment profiles
Source: Front Physiol. 2023 Apr 26;14:1121652. doi: 10.3389/fphys.2023.1121652 (PMC10169619; doi:10.3389/fphys.2023.1121652)
Supplement: Supplementary file 2 [file Image1.pdf]

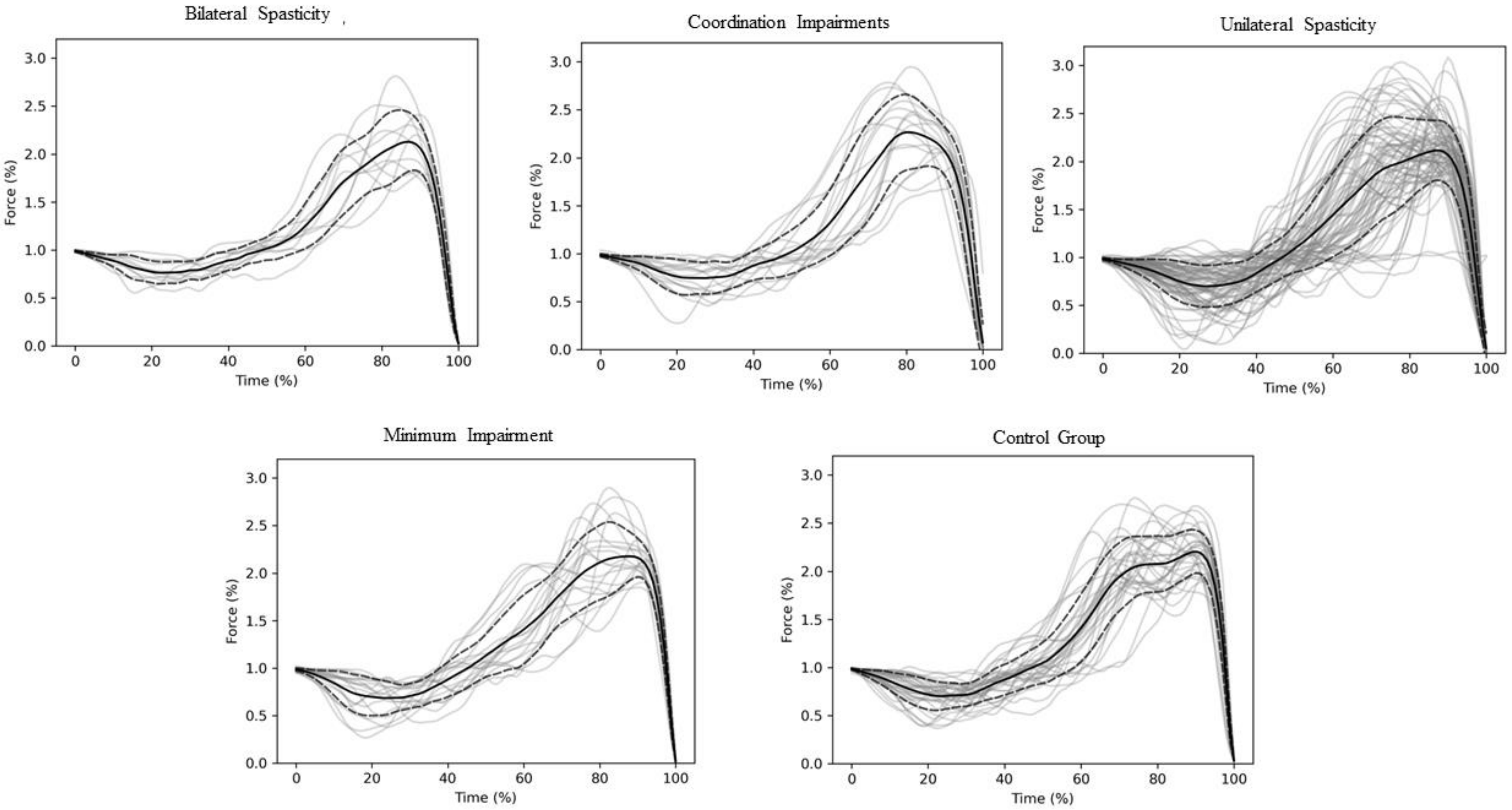

SUPPLEMENTARY FIGURE S1. The force-time curve of the countermovement jumps according to the impairment profiles of players with cerebral palsy and nonimpaired footballers.
